# CADM1 isoforms differentially regulate human mast cell survival and homotypic adhesion

**DOI:** 10.1007/s00018-012-0948-y

**Published:** 2012-03-22

**Authors:** Elena P. Moiseeva, Mark L. Leyland, Peter Bradding

**Affiliations:** 1 Department of Infection, Immunity and Inflammation, Institute for Lung Health, Clinical Sciences Wing, Glenfield Hospital, University of Leicester, Leicester, LE3 9QP UK; 2 Department of Biochemistry, University of Leicester, Leicester, UK

**Keywords:** Human, Mast cells, Adhesion molecules, Apoptosis, Allergy, Signal transduction, Lung

## Abstract

Cell adhesion molecule 1 (CADM1), expressed by human lung mast cells (HLMCs), mediates their adhesion to airway smooth muscle (ASM), and contributes to ASM-dependent HLMC proliferation and survival. CADM1 is expressed in alternatively spliced isoforms, but those present in HLMCs and their function are not known. We cloned three functional and one cryptic non-functional isoform with alternative splicing between exons 7/11 and 1/2, respectively, from HLMCs and human MC lines (HMC-1 and LAD2). Differentiated HLMCs and LAD2 cells expressed the functional isoform SP4 containing exons 7/8/11 (~80% of clones), as well as SP1 (exons 7/8/9/11) and a novel SP6 (exons 7/8/9/10/11). In contrast, immature HMC-1 cells expressed only functional SP4. SP4 overexpression in HMC-1 cells and HLMCs augmented homotypic adhesion to a greater extent than SP1 in various conditions. In contrast, CADM1 downregulation abolished homotypic adhesion, indicating that CADM1 is the sole receptor mediating mast cell aggregation. CADM1-mediated adhesion was enhanced by the presence of cell survival factors. SP1 overexpression in HMC-1 cells compromised survival compared to SP4 overexpression or control. CADM1 downregulation resulted in reduced viability and decreased expression of the pro-survival protein Mcl-1_L_, but not Blc-2 or Bcl-X_L_, and increased caspase-3/7 activity in both HMC-1 cells and HLMCs. This coincided with decreased basal Kit levels in HLMCs. In summary, human MCs express multiple CADM1 isoforms which exhibit differential regulation of survival and homotypic adhesion. The most highly expressed SP4 isoform is likely to contribute to MC aggregation and longevity in mastocytosis, and augment the pathophysiology of allergic diseases.

**Electronic supplementary material:**

The online version of this article (doi:10.1007/s00018-012-0948-y) contains supplementary material, which is available to authorized users.

## Introduction

Mast cells (MCs) play a critical role in the initiation of inflammation as a primary defence mechanism against pathogens, but their chronic activation also contributes to the pathophysiology of numerous diverse diseases including autoimmune disorders, allergy and asthma [1]. Human lung MCs (HLMCs) contribute to both inflammation and tissue remodelling in lung diseases such as asthma and pulmonary fibrosis [2], and their relocation within diseased lung tissue facilitates their interactions with structural cells such as airway epithelial cells, fibroblasts and airway smooth muscle (ASM) cells.

Adhesive interactions between cells are a fundamental mechanism of cell communication, permitting the accurate delivery of specific cell–cell signals. In constitutively adherent cell types, they are also critical for survival by regulating a specific form of apoptotic death, anoikis [3]. However, MCs, like other cells of haematopoietic origin, can survive and function in both adherent and suspended states. Cell adhesion molecule 1 (CADM1) is an immunoglobulin superfamily adhesion receptor which mediates adhesion through either homophilic or heterophilic molecular interactions. It is expressed on human MCs and mediates HLMC adhesion to ASM cells in a heterophilic manner; in turn, this direct contact drives HLMC proliferation and maintains their survival [4, 5]. In mouse^tg/tg^ MCs, heterophilic CADM1-mediated adhesion supports their survival in the peritoneal cavity and adhesion to fibroblasts [6, 7]. CADM1 therefore appears to play a key role in MC biology.

CADM1 is non-redundant in human physiology and has been implicated in several diseases including cancer, autism spectrum disorder and venous thrombosis [8–10]. CADM1 expression is significantly reduced in several cancers and inversely correlated with the disease progression [11–13]. In contrast, CADM1 is upregulated in acute T cell leukaemia and contributes to tissue infiltration [14, 15]. Human CADM1 was originally identified as a tumour suppressor of lung cancer 1 (TSLC1) [16]. Alternative splicing between exons 7 and 11 of the CADM1 gene is found in several species. Bioinformatics analysis of the CADM1 gene and cDNAs, conducted by the NCBI, Ensembl and by Biederer [17–19], identified alternatively spliced isoforms (SP) in several species with common SP4 (TSLC1, containing exons 7/8/11) and SP3 isoforms (containing exons 7/11), named here following Biederer [19]. In addition, cDNAs for human SP2 (with exons 7/9/10/11) and SP1 (exons 7/8/9/11) are present in DNA databases. Complicating the matter, CADM1 is a highly glycosylated protein (with a protein core of ~50 kDa) with N-glycosylation mapped to IgG domains (~25 kDa) and unmapped O-glycosylation (~25 kDa) [6, 20, 21]. Murine cultured MCs express SP4 and soluble CADM1 (sCADM1 with exon 7/intron 7) [22], but the isoforms expressed by human MCs and their individual roles are unknown.

Final MC differentiation is governed by the local environment within the tissue where MCs reside [23], whereas the majority of studies on MC physiology are conducted using MCs differentiated in cell culture. In addition, major phenotypic and functional differences are found between human and mouse mast cells [24]. The aim of this study was to characterise CADM1 expression and its role in the homotypic MC adhesion and survival in differentiated primary HLMCs and the neoplastic MC lines, HMC-1 and LAD2 [25, 26], derived from patients with MC leukaemia/sarcoma. We found that CADM1 is expressed as two major isoforms, SP4 and SP1, in differentiated human MCs and that these isoforms differentially regulate homotypic MC adhesion and survival.

## Materials and methods

### Cell culture

The human MC line HMC-1.1 (V560G) was cultured in Iscove’s Modified Dulbecco’s Medium (IMDM) with 10% foetal calf serum (FCS) as described previously [27]. HLMCs were isolated from normal lung obtained at surgery for carcinoma by selection using anti-CD117-coated Dynabeads [27]. The study was approved by the Leicestershire Research Ethics Committee and all participants gave written informed consent. Final MC purity was >99%. HLMCs were cultured in Dulbecco’s Modified Eagle’s Medium (DMEM), supplemented with 10% FCS, and 100 ng/ml stem cell factor (SCF), 50 ng/ml IL-6, and 10 ng/ml IL-10 as described previously [5]. Freshly isolated HLMCs were used for cloning and to study CADM1 expression. Stabilised 1- to 2-week-old HLMCs, adapted to cell culture, were used for adenoviral transduction.

### Primary antibodies

Anti-CADM1 3E1 IgY mAb was from Medical and Biological Laboratories, Japan. Anti-KIT (E1) mAb and anti-β-actin HRP-conjugated antibody were from Santa Cruz Biotechnology. Antibodies against KIT (mAb Ab81), Bim (mAb C34C5), Bcl-2 (mAb 50E3), Bcl-xL (mAb 54H6) and Mcl-1 (polyclonal #4572) were purchased from New England Biolabs.

### Protein analysis

Surface CADM1 expression was measured using FACS analysis of non-permeabilised cells. Cells were fixed in 4% paraformaldehyde in PBS and stained with anti-CADM1 mAb or control IgY, followed by FITC-conjugated anti-IgY Ab (Fisher Scientific) and then examined using a FACSCanto with FACSDiva2 software (BD Biosciences, USA). Since some CADM1 peaks were asymmetric, geometric means were used as a measure of the surface CADM1. Total protein analysis was conducted as previously described [28]. SDS-PAGE and immunoblotting were performed using the NuPAGE electrophoresis system (Invitrogen, USA). Blots were cut into horizontal strips with proteins of a close range of molecular weights, probed with appropriate antibodies. Alternatively, whole blots were sequentially probed with antibodies from different species. Protein bands on exposed films were quantified using a Syngene image system (Syngene, UK).

### Quantification of gene expression

All kits for RNA isolation and cDNA synthesis and Taqman probes (CADM1, β-actin, and 18S rRNA) were purchased from Applied Biosystems, USA. mRNA levels for CADM1, β-actin, and 18S rRNA were quantified using MX3000 thermocycler (Stratagene, USA) as previously described [28]. The relative levels of CADM1 expression were calculated by the 2^−Δ*CT*^ method [29].

### Cloning and analysis of CADM1 isoforms

Total RNA was isolated from the cell lines and HLMC specimens using RNA easy kit (Qiagen, UK). CADM1 cDNA was synthesised using AccuScript RT-PCR kit (Stratagene, USA) in RT-PCR with nested primers F1–R1 (all oligonucleotide primers are shown in Table 1), followed by primers F2–R2, and cloned into pSC-B plasmid using a Strataclone ultra blunt PCR kit (Stratagene, USA). Individual clones were isolated for each RNA source and analysed using several restrictases, including *BseR*1 and *EcoO*1091 with the sites within exons 8 and 10, respectively. All clones were analysed for alternative splicing between exons 7/11 and exons 1/2, using PCR with primers exon 7F/exon 11R and exon 1F/exon 2R, respectively. All DNA sequencing was done by the PNACL at the University of Leicester (http://www.le.ac.uk/mrctox/pnacl/). Sequences for CADM1 in bacterial clones p24 (SP4), p33 (SP1), p489 (SP6) and p15 (c15) have accession numbers HE586496, HE586497, HE586498, and HE586500, respectively, in EMBL–EBI (http://www.ebi.ac.uk/ena/home).

**Table 1:** List of oligonucleotides

Name	Sequence 5′→3′	Location in the CADM1 gene
F1	ggaggcagccaacgccgcca	5′UTR, exon 1
F2	ggacATGGCGAGTGTAGTGCTG	Overlapping initiation codon (ATG), exon 1
Exon 1F	CCGAGCGGATCCCAGTGT	Exon 1
Exon 2R	GTCGCAACCTCTCCCTCGAT	Exon 2
Exon 7F	CTGGGCCCAACCTGTTCATC	Exon 7
Exon 11R	AAGCACAGCATGGCGAACAC	Exon 11
R1	ggccagttggacacctcattgaa	Exon 12, 3′UTR
R2	ggctgat*CTA*GATGAAGTACTCTTT	Overlapping stop codon (*TAG*), exon 12
5′CADM1	ttcgaattcgacATGGCGAGTGTAGTGCTG	*Eco*RI site in 5′ to the initiation codon, exon 1
CADM1–5′GFP	ctcaccggtgAGATGAAGTACTCTTTCTTTTCTTC	*Age*I site in 3′ to codon 442, exon 12

### Modulation of CADM1 expression

SP4 cDNA was re-amplified using oligonucleotides 5′CADM1 and CADM1–5′GFP, cut with *Eco*RI and *Age*I restrictases and cloned in frame into *Eco*RI–*Age*I-cleaved pEGFP-N1 plasmid (Clontech, USA) to express CADM1–GFP-fusion protein. The fragments for CADM1 cDNAs, obtained by *Kpn*I–*Not*I restriction of respective plasmids, for SP4 (p24), CADM1–GFP (pCADM1-EGFP) and SP1 (p33) were recloned into a vector, provided by BioFocus (Netherlands, Leiden), and packed in Ad5C20Att01 adenovirus by BioFocus. Packaged adenoviruses were then purchased from BioFocus. All recloned cDNAs were sequenced to verify correct cloning. CADM1 sh RNAs (Sh3, Sh4, Sh5) were designed by BioFocus outside areas of alternative splicing. Luciferase shRNA (LucSh) was used as a control in downregulation experiments.

Adenoviruses containing SP4–GFP (see Fig. 5b) and GFP were used to optimise transduction conditions according to the manufacturer’s recommendations. Expression of CADM1–GFP or GFP in HMC-1 cells 6 days post transduction resulted in visible expression at variable levels in 70 ± 15 and 69 ± 8% of cells, respectively, as previously reported [30]. Similarly, transduction of various HLMCs with these viruses for 6 days produced stable and reproducible fluorescence. Transduction of HMC-1 cells and HLMCs with a multiplicity of infection of 50 IU/cell (17 × 3 IU/cell for mixed sh RNAs) for 6 days were used in all experiments, unless stated otherwise.

### Homophilic cell adhesion

Cells, dispersed as a single cell suspension, were cultured in 96-well plates for 3, 18 or 48 h in a cell incubator. Images were then captured on an Olympus CKX42 microscope (Olympus, UK). They were viewed and analysed using Cell^F^ imaging software (Olympus). The aggregates were outlined on an image and cross-sectional areas of the largest ten aggregates per photograph of a well were expressed in pixels^2^. The size of cell aggregates in every condition was estimated as the mean of the ten largest aggregates/well in four wells.

### Cell viability

Cell viability was examined by measuring cell number and caspase-3 activity as described previously [28]. Cell number was estimated using the ATPlite 1step kit (Perkin-Elmer, USA) using a calibration curve with a known number of HMC-1 cells, with luminescence detected in a plate reader. Caspase-3 activity was measured as accumulation of a specific fluorogenic substrate using the Apo-one Homogeneous Caspase-3/7 assay (Promega, USA), detected in a plate reader and expressed in fluorescent units/cell (FU/cell).

### Data analysis

All analysis was performed using GraphPad Prism 5 software (GraphPad Software, USA). All data are presented as the mean ± SE. Differences among the groups were analysed using a one-way ANOVA, followed by Dunnett’s test to determine whether the groups were different from a control group, or Bonferroni’s test to compare multiple groups. A repeated measures ANOVA was used to compare multiple data from the same donors. The *t* test was used to determine differences between two groups. Pearson’s and Spearman’s tests were used to analyse correlations. *P* < 0.05 was selected as the level of statistical significance.

## Results

### CADM1 is highly expressed in HMC-1 cells

**Fig. 1:**
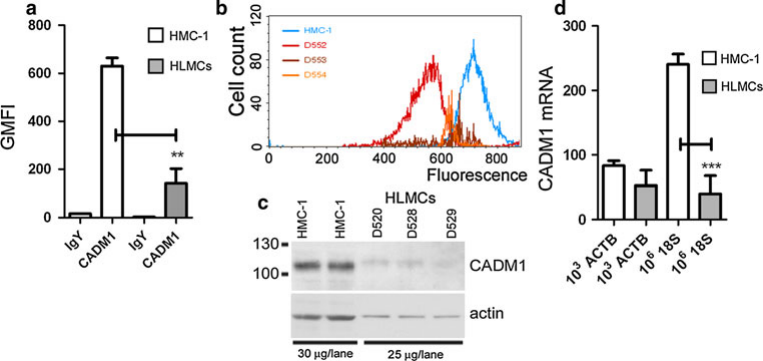
CADM1 is highly expressed in HMC-1 cells compared to HLMCs. Surface CADM1 expression is shown as a plot of geometric means (**a**) (*n* = 3, **P < 0.01) and a representative histogram for HMC-1 cells and HLMCs from donors D552–D554 (**b**). **c** Western blot of protein expression in HMC-1 cells and HLMCs from donors D520, D528 and D529. **d** Relative CADM1 mRNA levels are shown as a ratio of CADM1 mRNA molecules to normalisers [10^3^ actin B (ACTB) mRNA and 10^6^ 18S rRNA] (*n* = 3, ***P < 0.001)

Surface CADM1 expression was 4.5-fold higher in the HMC-1 cells compared to that in HLMCs (Fig. 1a, b). The total CADM1 protein content, as well as β-actin content, was also significantly higher in the HMC-1 cells (Fig. 1c). CADM1 mRNA expression was significantly higher in HMC-1 cells only when normalised to 18S RNA, but not to β-actin (ACTB) (Fig. 1d), which is consistent with the higher β-actin protein levels in HMC-1 cells compared to HLMCs.

### Several CADM1 isoforms are expressed in human mast cells

**Fig. 2:**
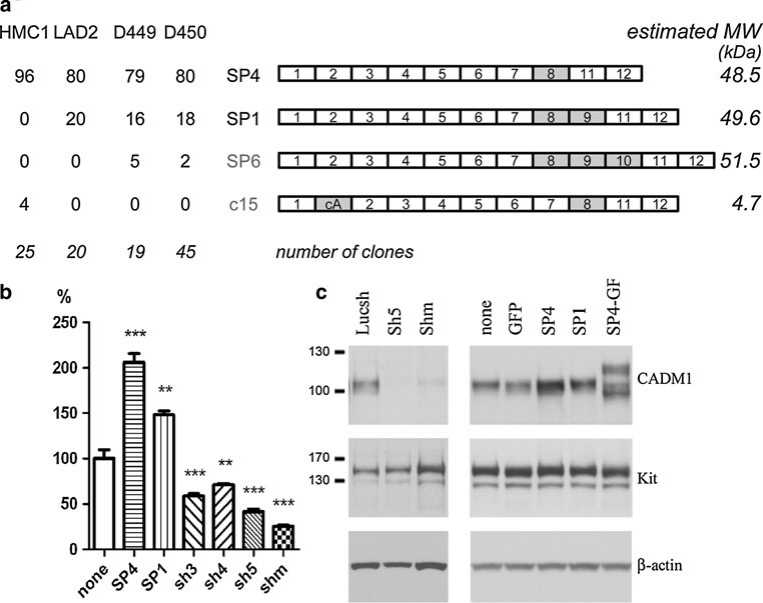
Multiple CADM1 isoforms are present in human mast cells. **a** The diagram summarises CADM1 isoforms cloned from HLMCs and cell lines with novel isoforms, SP6 and c15, shown in *grey*. The exons present in each clone are depicted not to scale. Alternatively spliced exons are coloured *grey*. The percentages of each isoform present among clones are shown for HLMCs, D449 and D450, and cell lines, HMC-1 and LAD2. The estimated molecular weight of each isoform is shown in kDa on the *right*. The numbers of analysed clones are shown in *italics*. For modulation of CADM1 expression, HMC-1 cells were transduced with adenovirus carrying SP4, SP1, SP4–GFP or CADM1 sh RNAs (*Shm* indicates mixed Sh RNAs). Luc sh RNA and GFP adenoviruses were used as controls. Surface and total CADM1 protein were detected by FACS (**b**) (*n* = 4, **P < 0.01, ***P < 0.001 compared to no virus) or western blotting (**c**) (representative of two experiments), respectively

First, we obtained full-length cDNA pools by RT-PCR from the HMC-1 and LAD2 cell lines and two HLMC specimens, D449 and D450 (donor identification number). These cDNA pools were cloned and plasmids from individual clones were analysed. All data are summarised in Fig. 2a. Restriction analysis of 109 independent cDNA clones revealed the presence of alternative splicing between exons 8 and 11. Sequencing of ten clones confirmed this and also identified alternative splicing between exons 1 and 2 in p15. Next, all plasmids were examined by PCR for alternative splicing between exons 7/11 and exons 1/2 (not shown). All clones contained exon 8. In addition to the SP4 isoform (exons 7/8/11), the differentiated LAD2 cells and HLMCs contained ~20% of the longer isoform SP1 (exons 7/8/9/11). A novel SP6 isoform (exons 7/8/9/10/11) encoded by all 12 exons of the CADM1 gene, which has not been described previously in humans or mouse, was cloned from HLMCs. In contrast, among 25 clones analysed from undifferentiated HMC-1 cells, 24 clones expressed the SP4 isoform and 1 clone, p15, contained an additional 86 nucleotides between exon 1 and 2, denoted as cryptic exon A in isoform c15 (Fig. 2a). This exon is located in the middle of the intron 1 and shifts a reading frame, inducing premature termination of CADM1 translation (an estimated molecular weight of translated protein for this and other isoforms is shown in Fig. 2a). The cryptic exon A is also present in partial CADM1 ESTs BD077366 and DA788329.

In order to investigate functions of individual isoforms, we used adenoviral delivery of SP4 and SP1, which represent the majority of CADM1 in differentiated HLMCs. Significant modulation of CADM1 expression was achieved by delivery of SP4, SP1 and sh CADM1 RNAs. Surface expression of SP4 and SP1 was increased up to 206 ± 10 and 148 ± 4%, respectively, compared to control HMC-1 cells (Fig. 2b). CADM1 Sh5 and mixed Sh RNAs markedly reduced CADM1 to 41 ± 3 and 25 ± 2% of control, respectively (Fig. 2b). CADM1 downregulation with both Sh5 and/or mixed Sh RNAs was used in further experiments. Western blotting confirmed that modulation of surface CADM1 was paralleled by changes in total CADM1 protein in transduced HMC-1 cells (Fig. 2c). SP1 consistently demonstrated a slightly higher molecular weight compared to SP4 [see Figs. 2c and 5c (below) at 0 h]. Furthermore, SP4–GFP expression influenced expression and glycosylation of endogenous CADM1 (Fig. 2c). No changes in the total amount of Kit or β-actin were observed. Similar CADM1 modulation was achieved in HLMCs [shown in Fig. 6b (below)].

### CADM1 mediates homotypic human mast cell adhesion

**Fig. 3:**
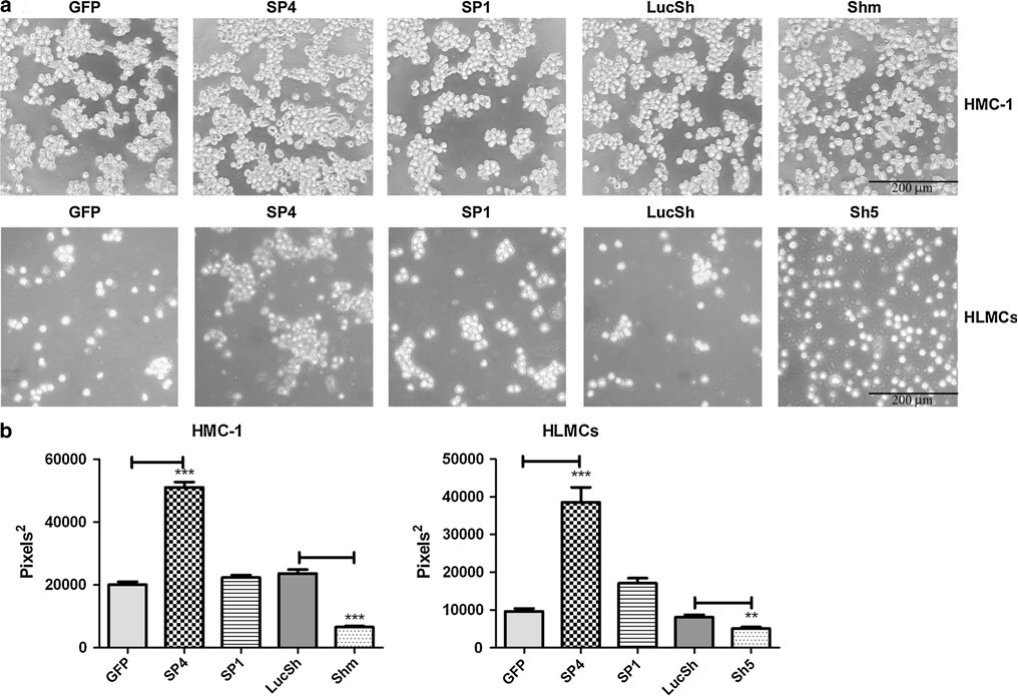
Modulation of CADM1 expression markedly affects homotypic mast cell–cell adhesion. **a** HMC-1 cells (*top panel* representative of two experiments) and HLMCs (*bottom panel* representative of one experiment with two pooled samples), were transduced with adenovirus carrying SP4, SP1 or CADM1 sh RNAs for 6 and 4 days, respectively. Luc sh RNA and GFP adenoviruses were used as controls. Transduced cells were resuspended as single cell suspension and incubated in growth medium for 3 h at 37°C. Original magnification ×100; each experiment was performed in quadruplicate. The cross-sectional areas of the largest aggregates were estimated on photographs of four wells for each condition (**b**) (*n* = 40, **P < 0.01, ***P < 0.001)

To examine the role of CADM1 in homotypic mast cell–cell adhesion, transduced HMC-1 cells were washed and then cultured for 3 h for analysis of cell aggregation. HMC-1 cells transduced with SP1, SP4 and control viruses (GFP and LucSh) formed cell aggregates from single cell suspension similar to non-transduced cells (not shown), whereas cells with downregulated CADM1 were present mostly as single cells (Fig. 3a, top panel). In order to estimate the sizes of cell aggregates, cross-sectional areas of the largest aggregates were compared. SP4-overexpressing cells formed larger aggregates compared to control HMC-1 cells (Fig. 3b) in agreement with published data for murine MCs [6]. In contrast, cells with downregulated CADM1 displayed reduced aggregation compared to controls (Fig. 3b).

Because SP4 overexpression in HLMCs led to the formation of large aggregates which maintained cell adhesion in growth medium even after multiple pipetting (Supplemental Fig. 1), HLMCs were transduced for a shorter time (4 days) to examine cell aggregation (Fig. 3a; Supplemental Fig. 2). Both SP4- and SP1-transduced HLMCs formed larger aggregates than control GFP-transduced HLMCs after 3 h (Fig. 3a, bottom panel). However, SP1 aggregates contained fewer cells than SP4 aggregates and were not statistically different from control aggregates (Fig. 3b). Sh5 RNA-transduced cells rarely formed aggregates, whereas control LucSh-transduced HLMCs formed occasional small aggregates, as did GFP-transduced cells (Fig. 3a, b). Hence, the SP4 isoform, in contrast to SP1, promoted fast cell–cell adhesion in both HMC-1 cells and HLMCs.

**Fig. 4:**
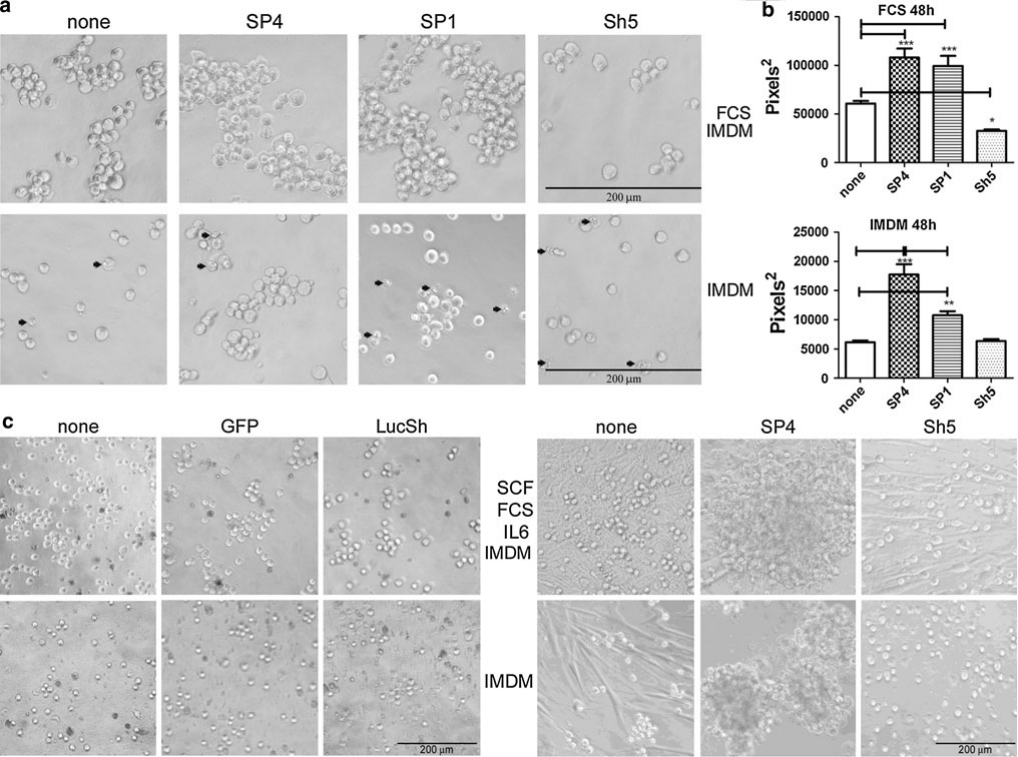
CADM1-mediated adhesion is influenced by CADM1 isoforms and survival signalling. **a** Transduced HMC-1 cells were cultured for 48 h in IMDM + 10% FCS (*top panel*) or in IMDM alone (*bottom panel*). *Arrows* indicate cells with membrane blebbing. Original magnification ×200; experiments performed in sextuplicate. The cross-sectional areas of the largest aggregates were estimated on photographs of four wells for each condition (**b**) (*n* = 40, *P < 0.05, **P < 0.01, ***P < 0.001). **c** HLMCs transduced with GFP and LucSh viruses (*left panel* representative of one donor, each assessed in quadruplicate) or SP4 and Sh5 adenoviruses (*right panel* representative of two donors, each assessed in quadruplicate) for 5 days, washed and grown for 72 h in 50% growth medium (50% HLMC medium/50% IMDM), or in IMDM alone. Original magnification ×100

Next, we examined cell aggregation over a longer time period (48 h) and in two culture conditions; normal medium (IMDM + 10% FCS) and IMDM in the absence of serum. Both SP4- and SP1-overexpressing HMC-1 cells formed larger aggregates than control cells after 48 h in both conditions (Fig. 4a, b). CADM1 downregulation reduced the size of aggregates only in IMDM + 10% FCS, because in the absence of serum non-transduced cells and cells with downregulated CADM1 did not form aggregates larger than two to three cells (Fig. 4a, b). In IMDM + 10% FCS, cells transduced with all viruses or control cells formed bigger aggregates compared to those in IMDM alone (Fig. 4a, b). When the cross-sectional data were examined by two-way Anova, both transducing viruses (*P* < 0.0001) and culturing conditions (*P* < 0.0001) influenced cell adhesion.

Aggregation of HLMCs was also studied. A diluted “50% growth medium” (50% HLMC medium/50% IMDM) was used to reduce the potential effect of proliferation. Transduction with either control GFP or LucSh viruses did not change cell aggregation (Fig. 4c). Non-transduced or control GFP/LucSh-transduced HLMCs adhered to plastic and formed small aggregates in “50% growth medium” after 72 h (Fig. 4c). The portion of HLMCs, which strongly adhered to plastic and spread on it, varied in HLMCs from different donors (compare left and right panels in Fig. 4c). SP4-overexpressing HLMCs formed larger aggregates compared to control cells in the same conditions (Fig. 4c) similar to SP4-overexpressing HMC-1 cells. In contrast, HLMCs with downregulated CADM1 were present mostly as single round cells and elongated cells adherent to plastic in “50% growth medium”, similar to control, but only as single round cells in IMDM alone. Paradoxically, CADM1 downregulation in some HLMCs appeared to reduce adhesion to plastic in IMDM, suggesting a crosstalk between CADM1 and other adhesion receptors. Altogether, our data suggested that CADM1 is responsible for homotypic cell adhesion of human MCs and CADM1-mediated adhesion is influenced by survival signalling in both HMC-1 cells and HLMCs.

β_2_-Integrin and ICAM-1 have been implicated in the homotypic adhesion of HMC-1 cells and human cultured MCs [31, 32]. Moreover, MCs express E-cadherin [33, 34], which may also contribute to aggregation. Hence, we examined MC aggregation in the presence of EGTA and EDTA to inhibit divalent cation-dependent aggregation via β_2_-integrins or cadherins. Neither EGTA nor EDTA affected aggregation of HLMCs cells after 3 h (Supplemental Fig. 2 top panel; EDTA data are not shown). SP4-overexpressing HLMC aggregates were not affected by incubation in EGTA or EDTA for 3 or 18 h; hence, neither integrins nor cadherins were involved in HLMC homotypic adhesion. HLMCs with downregulated CADM1 formed loose aggregates after 18 h only in the absence of EGTA/EDTA in contrast to non-transduced cells (Supplemental Fig. 2). Aggregation of control HMC-1 cells or cells transduced with SP4 was not affected by either EDTA or EGTA after 3 or 18 h (Supplemental Fig. 2 bottom panel). Thus, homophilic CADM1-dependent adhesion is exclusively responsible for homotypic cell–cell adhesion in MCs.

### SP1 over-expression and CADM1 downregulation compromise HMC-1 cell viability in the absence of survival factors

**Fig. 5:**
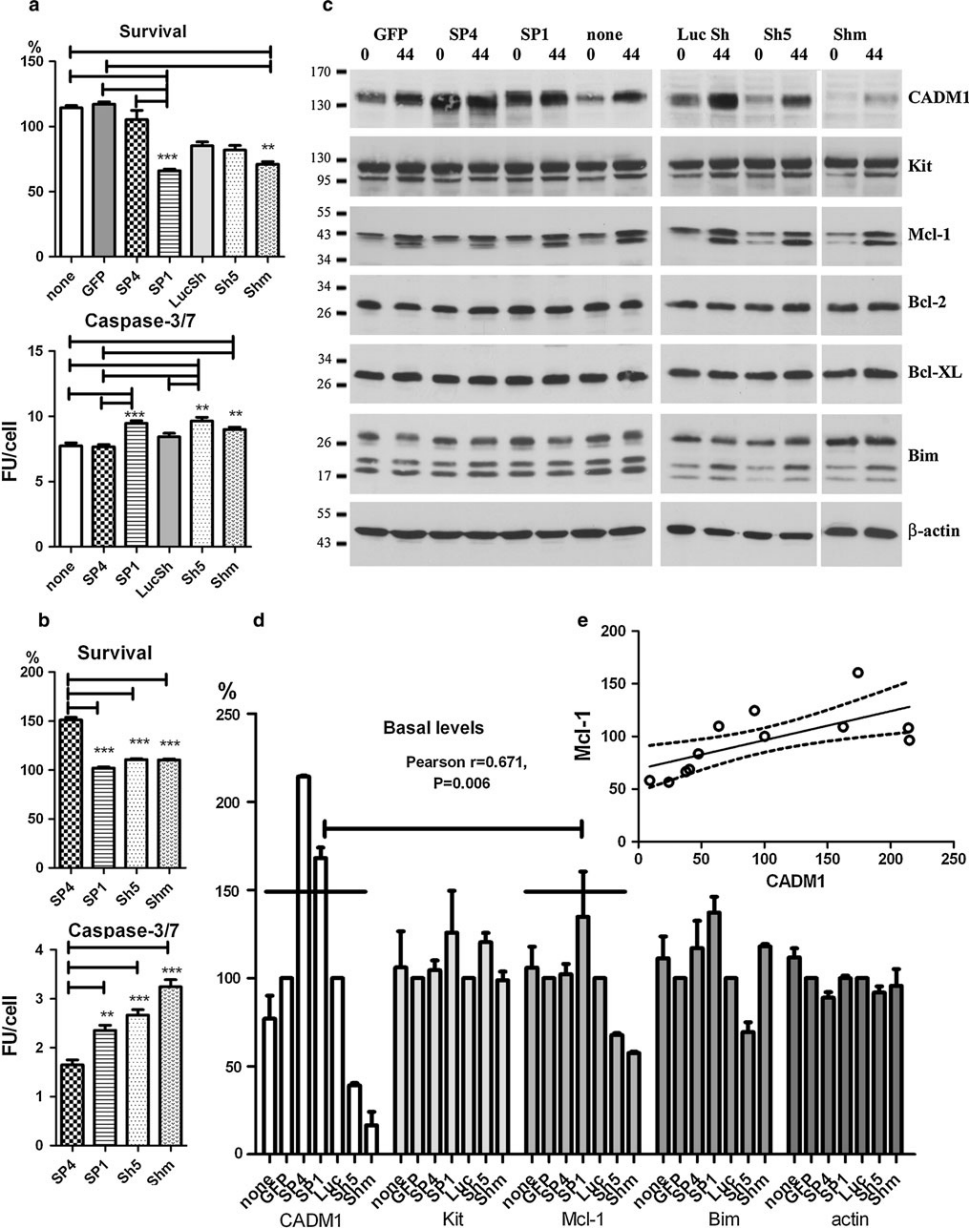
Modulation of CADM1 affects cell viability in HMC-1 cells. Transduced cells were washed and then incubated in IMDM alone for 48 h. The percentage of viable cells and caspase 3/7 activity (**a**) were measured in two experiments each performed in quadruplicate. Survival is shown as a percentage of cells surviving from the beginning of the experiment. **P < 0.01, ***P < 0.001. **b** Transduced HMC-1 cells were washed and incubated in IMDM with 1 μM A23187 for 24 h. Survival and caspase-3/7 activity were measured as in (**a**). Two experiments each performed in quadruplicate. **P < 0.01, ***P < 0.001 versus SP4. **c** Western blot of transduced cells at 0 and 44 h following removal of viruses in IMDM alone (representative of two to four experiments for different groups 30 μg/lane). Abs are shown on the *right*. **d** Protein bands, shown in (**c**) at a baseline (0 h) were quantified (*n* = 2). Bands in non-transduced, SP4 and SP1 groups, and non-transduced cells were expressed as percentages of GFP group; bands in Sh5 and Shm groups were expressed as percentages of LucSh group. Data for Bcl-2 and Bcl-XL are not shown. The data with 14 points for each protein (two experiments with seven groups each) were analysed for correlation. Regression analysis of these data is shown (**e**)

Transduced HMC-1 cells in the absence of serum displayed membrane blebbing, suggesting that they might become apoptotic (Fig. 4a). Viability was therefore investigated further. HMC-1 cells transduced with SP4 or control viruses survived to a similar degree as non-transduced cells in IMDM alone (Fig. 5a). In contrast, transduction with SP1 and Shm viruses decreased survival compared to SP4-overexpressing cells or control groups (Fig. 5a). These results indicate that the survival signal is not dependent on CADM1-dependent homotypic adhesion. The executioner caspase-3/7 activity was higher in CADM1-downregulated or SP1-overexpressing cells compared to SP4-overexpressing or control groups (Fig. 5a). Caspase activity could not be examined in GFP-expressing cells by this method. The TUNEL assay confirmed that CADM1 downregulation with mixed Sh RNA resulted in apoptosis with DNA breaks increased compared to control (not shown), but this assay was less sensitive than the caspase-3 activation assay. Next, we examined the effect of the calcium ionophore A23187 on the viability of cells with modulated CADM1. Again, SP4-overexpressing cells demonstrated better survival and lower caspase-3/7 activity compared to SP1-overexpressing or CADM1-downregulated cells (Fig. 5b).

Apoptosis-related protein expression was examined in HMC-1 cells deprived of serum. In particular, we were interested in Mcl-1 (myeloid cell leukaemia 1) and Bim (Bcl-2 interacting mediator of cell death) in regard to their roles in survival of mast cells in vivo and systemic mastocytosis [35–37]. After transduction for 6 days, protein levels were measured at baseline (0 h) and 44 h later in IMDM alone (Fig. 5c, d). CADM1 protein expression increased in control non-transduced, GFP- and LucSh-transduced cells 44 h after the media change (removal of serum), representing a possible compensatory response aimed at maintaining survival. CADM1 increased in Sh5/Shm-transduced cells due to re-expression of endogenous CADM1 after removal of transducing viruses (Fig. 5c). Pro-apoptotic Bim was upregulated at 44 h post removal of serum and viruses in some cells: particularly in Sh5-transduced cells, indicating the onset of apoptosis. Anti-apoptotic Mcl-1 was markedly increased at 44 h post removal of serum and viruses, and appeared as a strong doublet band, as described previously [35]; the size of the shorter Mcl-1 band (≥36 kDa) suggested that it was likely to be phosphorylated rather than alternatively spliced or cut by caspase-3 [38]. Modifications of Mcl-1 protein affect its function, such as protein stability and/or interactions with Bim [38]. Identification of the Mcl-1 shorter band was beyond scope of this study. Unexpectedly, when protein levels at the end of the viral transduction period (0 h) were compared between CADM1 downregulation and controls, there was a reproducible reduction of Mcl-1 to 68 ± 2 and 57 ± 1% in the Sh5 and Shm groups, respectively (Fig. 5d). Mcl-1 band densities in combined downregulation (Sh5 + Shm) were decreased compared to the combined control (none + LucSh) group (*P* < 0.001, *n* = 4). Furthermore, there was a correlation between CADM1 and Mcl-1 levels in HMC-1 cells with modulated CADM1 and regression analysis confirmed that CADM1 amounts predicted Mcl-1 levels in HMC-1 cells (Fig. 5d, e). Thus, several lines of evidence confirm a link between CADM1 and survival in HMC-1 cells.

### CADM1 downregulation compromises HLMC viability in the absence of survival factors and modulates expression of Kit and Mcl-1

**Fig. 6:**
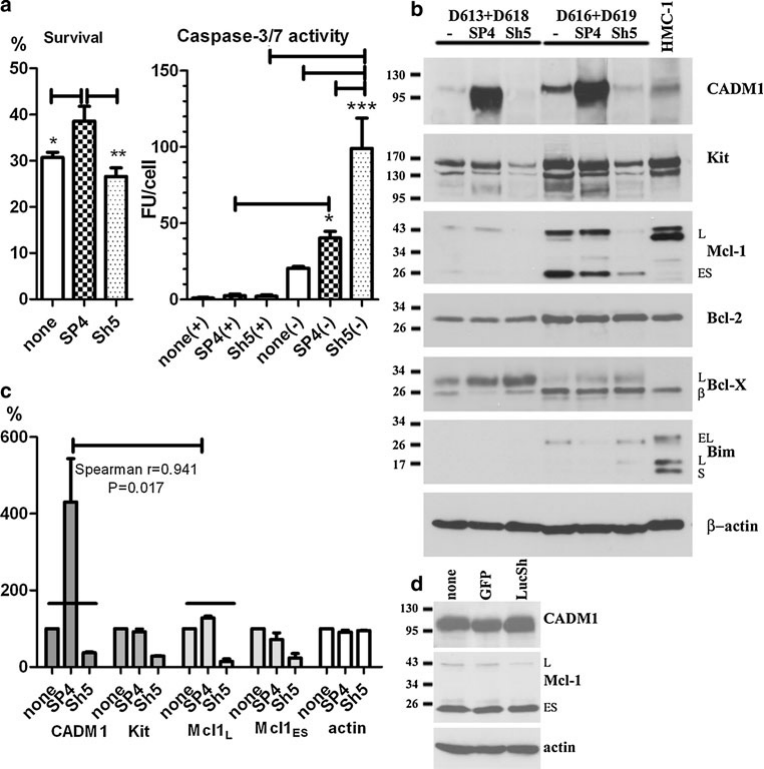
Modulation of CADM1 affects cell viability in HLMCs. **a** HLMC survival and caspase-3/7 activity were measured in transduced HLMCs after incubation for 72 h. All were measured in triplicate for each donor (*n* = 3). Survival in IMDM alone is shown as the percentage of cells present in 50% growth medium. Caspase 3/7 activity is shown in the presence (+) or absence (−) of growth factors. *P < 0.05, **P < 0.01, ***P < 0.001. **b** Pooled HLMCs from donors (D613 + D618) and (D616 + 619), transduced with SP4 or Sh5, were analysed by western blotting (25 μg/lane). Protein bands in (**b**) were quantified, normalised to non-transduced group and analysed for correlation (six points for each protein from two pools with three groups each) (**c**). **d** Western blot of proteins (25 μg/lane) from HLMCs D624, transduced with GFP and LucSh viruses

Next, we studied effect of CADM1 on survival of HLMCs in similar conditions to those above. HLMC survival in all conditions was considerably reduced in IMDM alone. Although SP4 overexpression in HMC-1 cells did not improve survival compared to controls, SP4-overexpressing HLMCs survived to a greater extent when compared to CADM1-downregulated or control cells (Fig. 6a). Incubation of HLMCs in IMDM alone for 3 days increased caspase-3/7 activity in SP4- and Sh5-transduced cells, from 3.9 ± 1.0 (50% medium) to 43.9 ± 4.3 FU/cell (IMDM alone) and 4.5 ± 1.0 (50% medium) to 108.9 ± 19.8 FU/cell (IMDM), respectively. Caspase-3/7 activity was drastically increased in CADM1-downregulated cells compared to SP4-overexpressing or non-transduced HLMCs (Fig. 6a). Hence, high CADM1 SP4 expression due to either high endogenous expression in HMC-1 cells, or following overexpression in HLMCs, improves mast cell viability compared to downregulation in the absence of other survival factors. These data are in agreement with those showing a protective effect of ectopic CADM1 expression on mouse^tg/tg^ MC survival in the peritoneal cavity [7].

HLMC specimens were pooled to obtain sufficient amounts of proteins for analysis. Analysis of two pools showed different isoforms of Mcl-1 and Bcl-X proteins, compared to HMC-1 cells, with isoforms indicated in Fig. 6b as published previously [39, 40]. In addition to the pro-survival Mcl-1_L_, HLMCs expressed the pro-apoptotic Mcl-1_ES_. Bim levels were very low in HLMCs and detectable only in one pool. CADM1 upregulation decreased pro-apoptotic Mcl-1_ES_ to 24 ± 16% in both pools and decreased Bim in one pool. CADM1 downregulation resulted in marked decreases in basal levels of Kit and Mcl-1_L_ to 29 ± 1 and 15 ± 9%, respectively, in both pools. No significant changes were observed in HLMCs transduced with control GFP or LucSh viruses (Fig. 6d). In addition, pro-apoptotic Bim_L_ was detected only in the Sh5-transduced pool of cells. Protein quantification showed a strong positive correlation between CADM1 and Mcl-1_L_ levels (Fig. 6c). Hence, CADM1 modulation induced visible changes in basal pro-survival/apoptosis protein levels. Overall, several lines of evidence indicated that CADM1 is an important survival factor in human MCs.

## Discussion

Much less is known about human CADM1 compared to mouse CADM1 despite its involvement in several diseases. For the first time, we have delineated the profile of CADM1 isoforms in human MCs, and the role of CADM1 in human MC homotypic adhesion and survival. Our data also revealed a link between CADM1 and Mcl-1, but not other Bcl-2 family proteins. In contrast to murine MCs which express only the SP4 and sCADM1 isoforms [6, 22], human MCs express several functional variants including SP4, SP1 and SP6, but not soluble CADM1 (not shown). In addition, we identified novel SP6 and non-functional cryptic c15 isoforms, which have not been described previously. Since murine MCs significantly differ from human MCs in many respects [24], it is possible that CADM1 expression may contribute to some of the observed phenotypic differences.

Although the SP4 isoform is prevalent in all human MCs, the longer SP1 and novel SP6 isoforms were found only in highly differentiated HLMCs. Interestingly, all cloned isoforms contained exon 8, encoding 17 Thr codons out of a total of 28. The NetOGlyc 3.1 server [41] predicts that the only O-glycosylation sites in CADM1 are all 17 Thr in exon 8 and 4 Thr in exon 9. In agreement with the data on CADM1 glycosylation, the SP4 protein core is ~50 kDa, but it runs at about 105 kDa in western blots and is expected to be both N- and O-glycosylated. Considering that differences between SP4 and SP1 do not exceed ~4–8 kDa (1.1 kDa for amino acid residues encoded by exon 9 + four additional O-glycosylation units with molecular weight about 3.2–6.4 kg/mol [42]), these isoforms are not easily separated in SDS-PAGE. Similarly, SP6 is just ~1 kDa heavier than SP1. Supporting the prediction, overexpressed SP1 had a slightly higher molecular weight compared to SP4 in western blots. The significance of CADM1 O-glycosylation is not established, but it is feasible that glycosylated threonines form a stiff stalk (2.5 Å per each glycosylated residue [41]) in the extracellular domain which moves the first IgG domain, involved in cell adhesion, further away from cell membrane. This extension might facilitate adhesion, which could be important for interactions of HLMCs with structural airway cells.

**Fig. 7:**
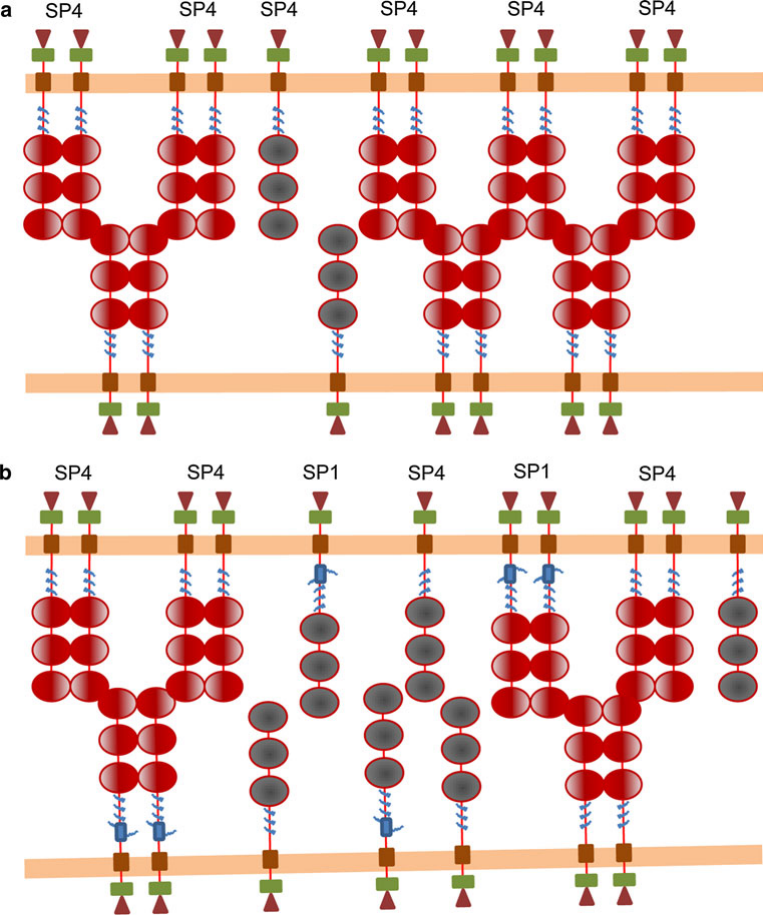
A model of adhesion mediated by SP4 and SP1. CADM1 monomers, shown as *grey *molecules, are inactive in adhesion. When cells adhere, CADM1 forms dimers, shown as *red* molecules, which are actively involved in adhesion. **a** Two cells express only SP4, which forms active dimers involved in cell adhesion. **b** Two cells express both SP4 and SP1 (molecule with additional protein sequence encoded by exon 9, shown as a *blue box* in SP1 stalk near cell membrane) with the same density along cell membrane (*orange line*). Because SP4 and SP1 extracellular domains have different lengths, they cannot align properly and form dimers. The total number of CADM1 dimers, involved in cell adhesion, is reduced on the cell surface. However, over time, most of the CADM1 molecules would be able to find a matching partner to dimerise with and cells would be able to adhere as strongly as cells expressing only one CADM1 isoform

SP4 and SP1 displayed differences in function in terms of adhesion and survival. Both isoforms promoted aggregation in some conditions, but SP4 overexpression promoted fast (within 3 h) cell aggregation of both HLMCs and HMC-1 cells, whereas SP1 increased aggregation only after prolonged incubation (48 h). To explain these facts, we propose the model illustrated in Fig. 7. Since SP1 has a longer extracellular domain, it is possible that it cannot align the immunoglobulin domains with those in SP4 and, as a result, does not form dimers with SP4. Hence, cells expressing only the SP4 isoform would form CADM1 dimers on the cell surface faster than cells expressing both SP4 and SP1 with the same CADM1 density on the cell surface. Since CADM1 dimers are involved in cell adhesion, cells expressing only SP4 would have more functional CADM1 dimers on the surface and would be able to adhere faster. However, cells expressing both SP4 and SP1 would form more SP4/SP4 and SP1/SP1 dimers over a longer period of time, which would provide strength of adhesion similar to that in cells expressing SP4 only.

Homotypic adhesion of human MCs is suggested to be mediated by β2-integrins [31, 32]. In our experiments, there was little evidence for integrin-dependent homotypic adhesion. In contrast, downregulation of CADM1 both in HLMCs and HMC-1 cells markedly reduced homotypic MC adhesion in normal growth medium and abolished it in the absence of survival factors. CADM1 is, therefore, a key receptor mediating homotypic adhesion in both leukaemic MCs and differentiated HLMCs. The strength of CADM1-mediated adhesion was influenced by other factors such as growth conditions and cell type (cells in SP4-transduced HLMC aggregates did not separate after 6 days, but did in HMC-1 aggregates), suggesting that cross-signalling plays a role in CADM1-dependent adhesion. In addition, interactions between CADM1 and Kit may influence adhesion [5]. A connection between CADM1 and Kit was particularly obvious in HLMCs with downregulated CADM1 (Fig. 6b), where a reduction in CADM1 was paralleled by a reduction in Kit.

Abnormal accumulation of MC aggregates in various tissues is characteristic of advanced mastocytosis and MC accumulation in bone marrow on the background of systemic mastocytosis is a major diagnostic factor for MC leukaemia [43]. Leukaemic HMC-1 cells express 4.5-fold more CADM1 than HLMCs, suggesting that CADM1 may be involved in MC aggregation in this disease. Furthermore, the D816V KIT mutation present in the majority of patients with mastocytosis is not adequate to induce aggressive MC disorders in isolation [44]. We propose that upregulation of CADM1 SP4 may be an important co-factor. This raises the possibility that MC leukaemia is analogous to T cell leukaemia with high CADM1 levels required for malignant transformation and tissue infiltration [14, 15].

In support of the above hypothesis, the effect of CADM1 on survival was striking both in HLMCs and in HMC-1 cells. SP4 overexpression did not affect survival or caspase-3/7-activity in HMC-1 cells, but increased survival in HLMCs, indicating that the relatively high CADM1 SP4 only levels in leukaemic HMC-1 cells are already sufficient to impart a survival benefit. Conversely, CADM1 downregulation reduced survival, increased caspase activity and decreased Mcl-1, which is a survival factor for MCs [35, 36] in both HLMCs and in HMC-1 cells, suggesting a critical role in MC survival. Thus, reduced viability in cells with downregulated CADM1 may be caused by reduced expression of Mcl-1, which would affect the balance between pro-survival and pro-apoptotic Bcl-2 family proteins impinging on cell viability [45]. The levels of caspase-3/7 activity upon withdrawal of survival factors in HLMCs were considerably higher than those in HMC-1 cells, indicating that these leukaemic cells have high anti-apoptotic signalling. In addition, there were striking differences between HMC-1 cells and HLMCs in basal levels of proteins involved in cell survival, such as Mcl-1, Bcl-X and Bim. Moreover, the differences were not only in the levels of expression, but in the spectrum of expressed isoforms.

In addition, SP1 overexpression reduced HMC-1 survival, which implies that it may exert dominant-negative activity over SP4, and may explain why HMC-1 cells only express functional SP4. Interestingly, leukaemic LAD2 cells, expressing both SP4 and SP1, are less robust, proliferate slowly, and require additional factors for growth and survival [46]. In the case of SP1, we did not find specific changes in Mcl-1 expression or any others to indicate the mechanisms of reduced survival in SP1-overexpressing MCs. It has been suggested that the presence of exon 9 in CADM1, which is present in SP1, results in constitutive shedding of the CADM1 extracellular domain and an increased presence of the intracellular domain within cells [47]; this is likely to impact on cell signalling and may affect CADM1-dependent survival.

Although the SP4 CADM1 isoform promotes both MC homotypic adhesion and survival, these two processes do not appear to be directly related. In the absence of serum, control non-transduced HMC-1 cells do not aggregate, but still survive to a similar degree as SP4-transduced cells which form large aggregates. In contrast, SP1-overexpressing cells do aggregate but survive to a lesser degree than control cells, and CADM1 downregulated cells which do not aggregate have impaired survival. It is therefore possible to have several scenarios: increased SP1-dependent adhesion with reduced survival, maximal adhesion in SP4-overexpressing cells with maximal survival, medium strength adhesion with maximal survival in constitutive SP4-expressing HMC-1 cells, and minimal adhesion in CADM1-downregulated cells with reduced survival. This suggests that CADM1 SP4 and SP1 deliver survival signalling independently of their role in cell aggregation and that CADM1-dependent MC survival does not appear to be dependent on CADM1-dependent adhesion. In this respect, CADM1 is similar to other immunoglobulin-like CAMs (cell adhesion molecules), such as NCAM, which can activate signal transduction cascades in the absence of cell adhesion [48].

Our data show that CADM1 plays a key role in MC aggregation and survival and, therefore, is likely to contribute to both clonal expansion and aggregation of MCs in advanced mastocytosis. Our data indicate that SP4 is a survival factor for MCs, and it is therefore likely to be of particular importance for MC survival when other survival signals are unavailable. HLMCs, as a first line for immune defence against infections and pathogens, are characterised by a fast response to the changing environment and relocation into different lung compartments in disease [2]. CADM1 may provide anti-apoptotic signalling during these physiological changes, which involve disassembly of survival signalling during cell migration and relocation. CADM1 may also contribute to the longevity of MCs and, thereby, augment pathology of disorders such as asthma and allergy.

CADM1 is an attractive target for the modulation of MC-dependent disease. Targeting CADM1-dependent adhesion directly may not be a useful therapeutic approach because of its importance in other cell types, but CADM1-induced signalling is likely to be specific to MCs and a more appropriate target for intervention. For example, the CADM1 partners, TIAM in acute T cell leukaemia [49] or PTPN13 in epithelial cells [50], are not expressed in MCs. Also, no link between CADM1 and Mcl-1 has so far been reported. The molecular mechanisms of the CADM1-mediated pro-survival pathway are not known, but by delineating isoform-specific expression and intracellular signalling within different cell types, there is the potential to modulate cell and tissue-specific CADM1-dependent processes favourably.

## Electronic supplementary material

Below is the link to the electronic supplementary material.

Supplementary material 1 (PDF 89 kb)

Supplementary material 2 (PDF 427 kb)

## Abbreviations

ASM: Airway smooth muscle
CADM1: Cell adhesion molecule 1
HLMCs: Human lung mast cells
MCs: Mast cells
SP: Alternatively spliced isoform
TSLC1: Tumour suppressor of lung cancer 1
