# Supplementary material for: CADM1 isoforms differentially regulate human mast cell survival and homotypic adhesion
Source: Cell Mol Life Sci. 2012 Mar 22;69(16):2751–64. doi: 10.1007/s00018-012-0948-y (PMC3400039; doi:10.1007/s00018-012-0948-y)
Supplement: Supplementary file 1 — Supplementary material 1 (PDF 89 kb) [file 18_2012_948_MOESM1_ESM.pdf]

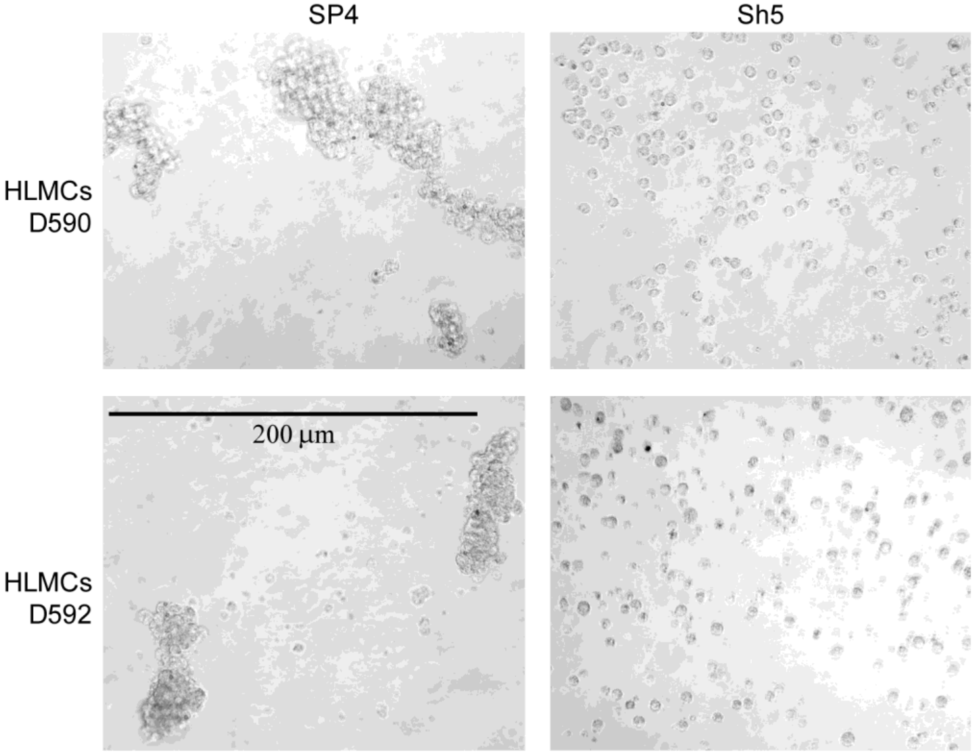

Supplemental figure 1. SP4 overexpression results in strong cell adhesion in HLMCs. HLMCs, D590 and D592, were transduced with SP4 and Sh5 RNA, washed with HBSS and pipetted 5 times. Magnification x100.
