# Supplementary material for: CADM1 isoforms differentially regulate human mast cell survival and homotypic adhesion
Source: Cell Mol Life Sci. 2012 Mar 22;69(16):2751–64. doi: 10.1007/s00018-012-0948-y (PMC3400039; doi:10.1007/s00018-012-0948-y)
Supplement: Supplementary file 2 — Supplementary material 2 (PDF 427 kb) [file 18_2012_948_MOESM2_ESM.pdf]

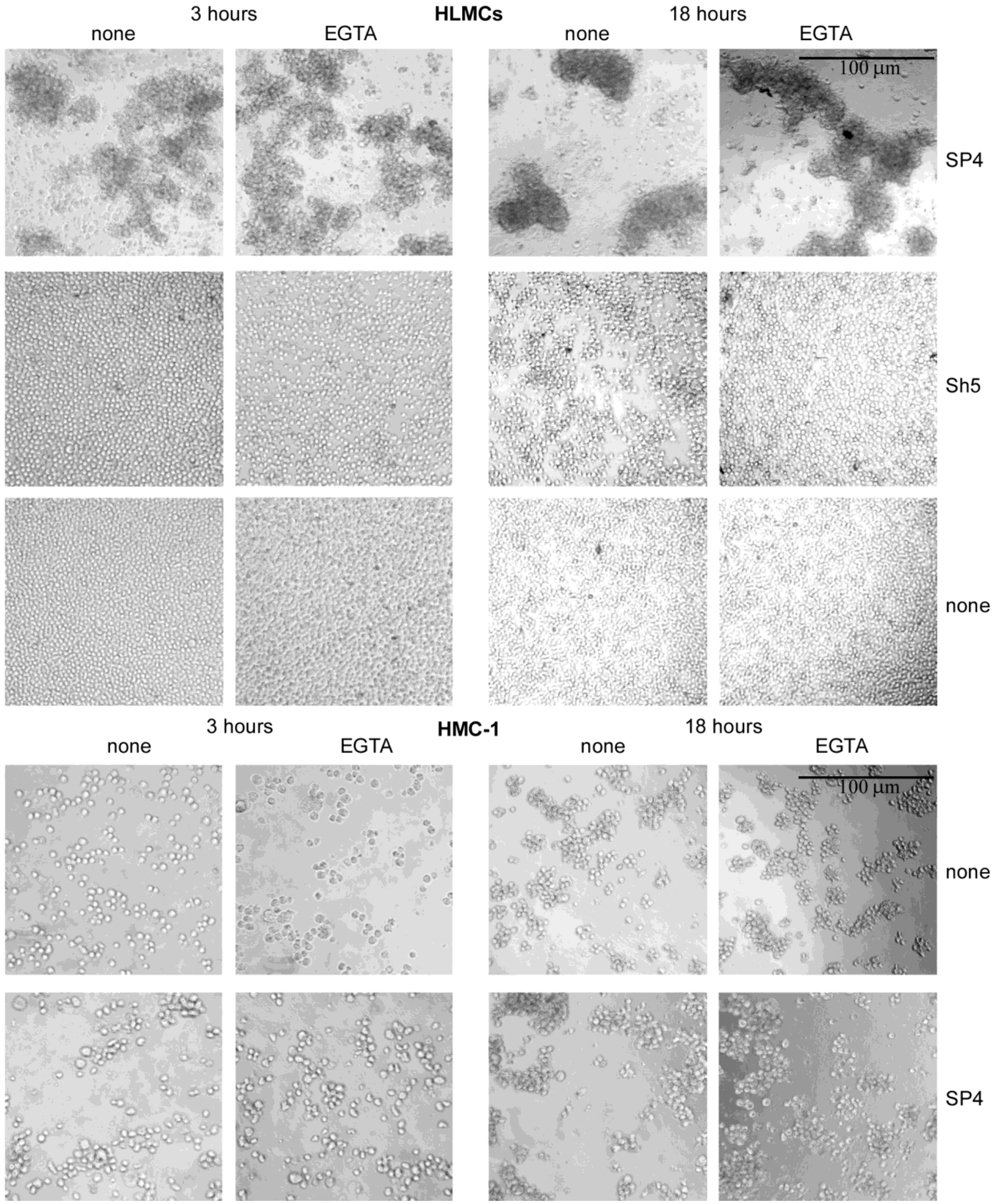

Supplemental figure 2. Homotypic mast cell adhesion is mediated by CADM1. HLMCs D613 (top panel) were transduced with SP4 and Sh5 RNA, washed with HBSS, resuspended in full medium in the absence or presence 5 mM EGTA and incubated for 3 and 18 hours. Original magnification x200; representative of 6 wells. HMC-1 cells (bottom panel) were transduced with SP4, washed with HBSS, resuspended in full medium in the absence or presence 5 mM EGTA and incubated for 3 and 18 hours. Original magnification x200; representative of an experiment performed in sextuplet.
